# Target Recognition of Industrial Robots Using Machine Vision in 5G Environment

**DOI:** 10.3389/fnbot.2021.624466

**Published:** 2021-02-25

**Authors:** Zhenkun Jin, Lei Liu, Dafeng Gong, Lei Li

**Affiliations:** ^1^Department of Information Engineering, Wuhan Business University, Wuhan, China; ^2^Graduate School, Gachon University, Seoul, South Korea; ^3^Department of Information Technology, Wenzhou Polytechnic, Wenzhou, China; ^4^Huawei Technologies Co. Ltd., Shenzhen, China

**Keywords:** machine vision, artificial intelligence, deep learning, industrial robot, 5G environment

## Abstract

The purpose is to solve the problems of large positioning errors, low recognition speed, and low object recognition accuracy in industrial robot detection in a 5G environment. The convolutional neural network (CNN) model in the deep learning (DL) algorithm is adopted for image convolution, pooling, and target classification, optimizing the industrial robot visual recognition system in the improved method. With the bottled objects as the targets, the improved Fast-RCNN target detection model's algorithm is verified; with the small-size bottled objects in a complex environment as the targets, the improved VGG-16 classification network on the Hyper-Column scheme is verified. Finally, the algorithm constructed by the simulation analysis is compared with other advanced CNN algorithms. The results show that both the Fast RCN algorithm and the improved VGG-16 classification network based on the Hyper-Column scheme can position and recognize the targets with a recognition accuracy rate of 82.34%, significantly better than other advanced neural network algorithms. Therefore, the improved VGG-16 classification network based on the Hyper-Column scheme has good accuracy and effectiveness for target recognition and positioning, providing an experimental reference for industrial robots' application and development.

## Introduction

In the early 1960s, the United States was the first country in the world to manufacture industrial robots. After that, industrial robot technology and its products developed rapidly. At present, industrial robots have provided productivity tools for automation in various industries (Li et al., 2018). At the same time, the growing use of artificial intelligence technology in industrial robots that have been widely used has dramatically changed the way people produce and live.

An industrial robot is a machine that automatically performs industrial production tasks. It can accept commands, perform corresponding functions according to programming procedures, or operate according to the principles of artificial intelligence technology. Its main applications include plundering, assembly, acquisition, placement, product inspection, and testing. The ability of industrial robots to perform these functions relies on accurate detection and identification of targets (Dönmez et al., 2017; Fernandes et al., 2017; Song et al., 2017; Zhang et al., 2017). The basis for realizing the above functions is the industrial robot vision system, which realizes the visual function of the robot through a computer, enabling the robot to recognize the objective world. Therefore, the vision system plays an important role in improving the performance of industrial robots. Industrial robot vision systems are used for target detection and task identification. Image feature extraction technology is the basis of machine vision. It refers to the extraction of certain key information that can be used to represent images from image data for machine vision tasks such as subsequent recognition and classification (Ertuğrul and Tağluk, 2017; Sung et al., 2017; Wang et al., 2018) With the rapid development of communication technology, artificial intelligence, and machine learning, the precondition of the 5G environment has matured. The new generation of wireless communication technology not only means faster transmission speed but also better security performance for industrial robots (Bogue, 2017). The application of these intelligent algorithms to the development of industrial robots with higher service levels will bring new opportunities for the development of industrial robots.

However, a large amount of existing research is at the level of simple industrial devices. With the diversification of industrial devices, the traditional positioning and recognition algorithm has the problems of serious positioning errors, slow recognition speed, and low accuracy, which leads to the fact that the sorting work in complex environments is still in the manual operation stage. Therefore, to improve the accuracy of industrial robots for object recognition and positioning in complex environments, a visual recognition and localization algorithm based on artificial intelligence deep learning is proposed. The deep learning network model is used to convolve and pool the image layers, and the target classification algorithm is used to optimize the industrial robot visual recognition system. Besides, the Fast R-CNN algorithm and the improved VGG-16 classification network based on the Hyper-Column scheme can identify and classify the target objects in a complex background. The simulation results have shown that the proposed algorithm has good stability and accuracy.

## Literature Review

### The Development Trend of Deep Learning

With rapid science and technology development, artificial intelligence (AI) has been applied in more fields. As a new intelligent algorithm in the 21st century, DL has been studied by many scientific researchers. In 2006, Hinton, the expert in the field of artificial intelligence of the University of Toronto, proposed the concept of deep learning. He proposed an algorithm for fast training of deep neural networks, which opened up the craze for deep learning in the field of artificial intelligence (Hinton et al., 2017). Deep learning mathematical models generally include deep convolution neural networks using supervised learning methods, and superposition self-coding networks and deep belief networks using hybrid supervised learning methods. Stani et al. (2017) proved that the deep learning algorithm has superior non-linear approximation ability and generalization ability than BP neural network, support vector machine (SVM), and other network models, and can exhibit very powerful performance in complex pattern recognition occasions. To overcome the limitations of existing hybrid precoding schemes, Huang et al. (2019) proposed a mmWave massive MIMO hybrid precoding framework based on deep learning, which regarded each selected pre-coder as the mapping relationship in Deep Neural Network (DNN). Simulations found that the DNN-based method could minimize the bit error rate and improve the spectrum efficiency of mmWave's massive MIMO. While significantly reducing the computational complexity, hybrid precoding could provide better performance than traditional schemes. Sun et al. (2020) proposed an adaptive Deep Learning-aided Digital Pre-Distorter (DL-DPD) using optimized deep Recurrent Neural Networks (RNN). The experimental results proved the effectiveness of the proposed adaptive DL-DPD and revealed that the online system switched sub-DPD modules more frequently than expected.

### Research of Target Recognition Technology

Traditional detection and recognition technologies include segmentation-based method, feature analysis method, image recognition, and decision-classification method, and pattern learning and shape matching method, which are widely used in the industrial fields. With AI technologies' development, such as machine learning, the target recognition accuracy has attracted many scholars' attention. You et al. (2017) built a SCARA robot automatic recognition and positioning plug-in system platform based on monocular vision. Through the calibration of the crawling system parameter model and the establishment of camera parameters, the color recognition of the workpiece and the position information of the workpiece could be achieved. Besides, the robot's jaws could be controlled to accurately grasp the target workpiece, meeting the real-time requirements of grasping the workpiece in general industrial production. Sampedro et al. (2019) proposed a fully autonomous aerial robot scheme for performing complicated Search And Rescue (SAR) tasks in an unstructured indoor environment. The algorithm integrated a new type of deep reinforcement learning method to identify and interact with targets in multiple environments. Wang et al. (2020) proposed a small humanoid combat robot design scheme based on the target recognition algorithm for the low recognition rate of mobile robots in complicated working environments. This scheme could fuse information by adding visual information, which was simpler and ran faster and more effectively. Finally, the simulation experiment proved its effectiveness. Li et al. (2020) investigated the control performance of the visual servo system under planar cameras and RGB-D cameras. They segmented the color images collected by RGB-D cameras based on optimized normalized cut sets. A control cycle could be completed by calculating the end angle and speed of the robot, and the entire process was iterated until the servo task was completed. Finally, experiments verified that the proposed RGB-D image processing algorithm had an excellent performance in the above aspects of the visual servo system.

According to the above works, the DL algorithm is applied in many fields, and its application in industrial robot target recognition is scarce. Therefore, based on the DL algorithm, CNN is improved based on the Hyper-Column scheme. The constructed algorithm's performances are analyzed to study industrial robot target recognition, which has significant research value (Wen, 2020).

## Proposed Method

### Target Detection Technology Based on Deep Learning of Artificial Intelligence

**Figure 1:**
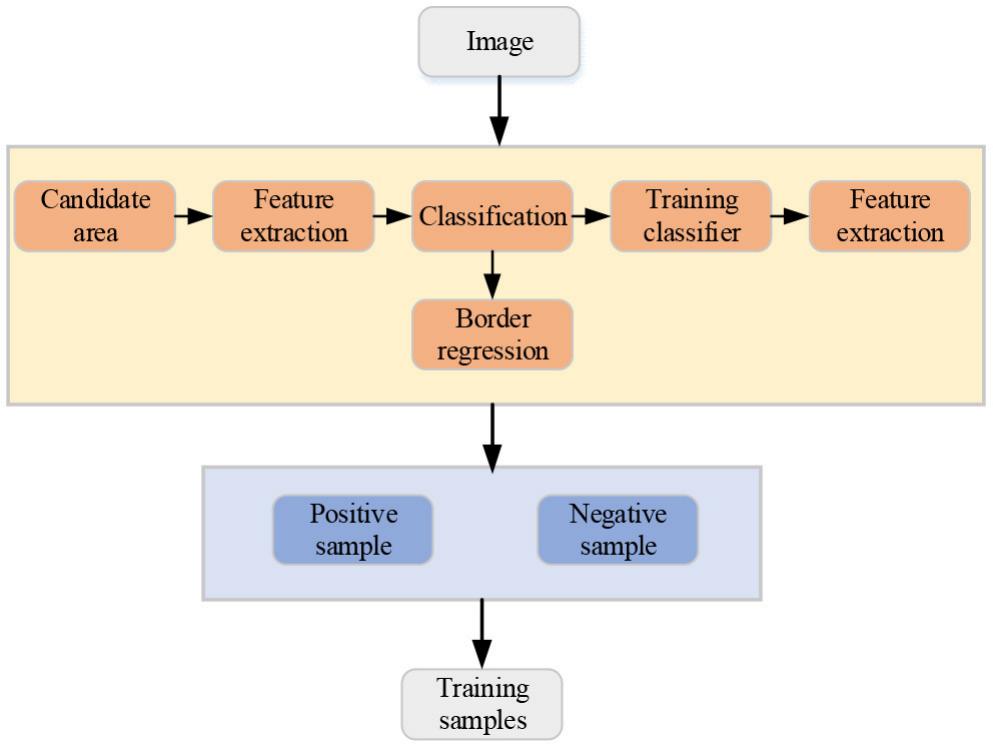
The basic flow chart of target detection technology.

Deep learning (DL) is one of the technical and research fields of machine learning. By establishing an artificial neural network with a hierarchical structure, artificial intelligence is realized in the computing system. The artificial intelligence target detection technology of images refers to the application of techniques such as machine vision to determine the type, position, size, and confidence of the target object, and the predetermined target object is automatically detected from the image (Wang et al., 2019). The basic process of target detection technology is shown in Figure 1.

Researchers have proposed a Fast Region Convolution Neural Network (Fast R-CNN) detection method in recent years, which transfers feature extraction to the convolution feature map of the last layer (Matson et al., 2019). The region convolution neural networks (CNN) need to repeat the problem of multiple convolution calculations for the same image; at the same time, the discrimination of the proposed area and the bounding box regression are integrated into one framework, which effectively improves the accuracy and efficiency of target detection. Fast R-CNN uses a single network joint training convolution neural network, classifiers, and bounding box regenerators to extract image features (CNN), classification (SVM), and compact bounding boxes (regressors). The Fast R-CNN network takes the entire image and a set of candidate frames as input, first using several convolution layers and a maximum pooling layer to process the entire image, producing a convolved feature map, and then for each candidate box, the pooling layer of regions of interest extracts a fixed-length feature vector from the feature map. Each feature vector is fed into a series of fully connected layers, which are finally branched into two output layers; one outputs k object categories and the probability estimation for a background category, and the other one outputs four real values for k object categories. Fast R-CNN solves many problems of R-CNN with faster training speed; however, there are still many problems. Fast R-CNN uses the Selective Search algorithm to extract the candidate regions, while the target detection consumes a lot of time. The candidate region extraction takes 2~3 s, and the single image detection time reaches 0.32 s. The extraction efficiency of the recommended region is low, which still cannot meet the demand of the real-time application.

### Convolution and Pooling of Image Layers by the Artificial Intelligence-Based Deep Learning Network Model

**Figure 2:**
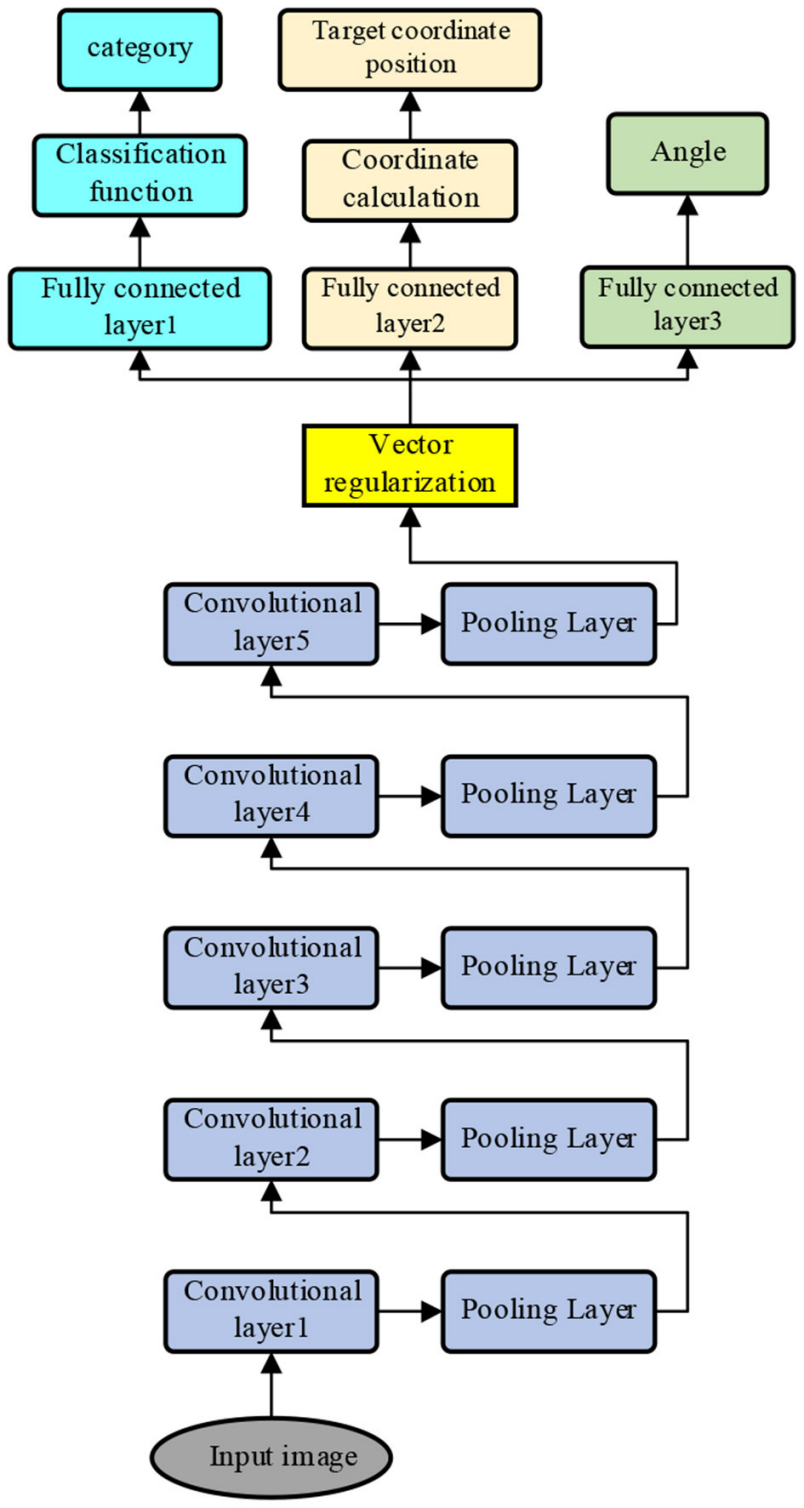
The model of layered convolution kernel pooling of deep learning network.

The deep learning network receives the recorded visual feedback image in real-time and performs deep learning processing on each frame of the received image, i.e., the multi-level convolution, pooling operation, and classification processing to obtain the coordinates, angles, and time of the detection target on the image in the image coordinate system, focuses on the coordinates and angle of the detection target on the captured visual feedback image, and sends the processed image and the coordinates, angles, and time information of the detection target on the image to the intermediate result synthesis processing unit; the processed image sent to the human error correction interface is shown in Figure 2.

The time mentioned in the above content refers to the image capturing time, the angle is the angle between the target re-axis and the image coordinate system X; the image coordinate system describes the position coordinates of a single-pixel in the whole image, and the pixel point in the upper left corner of the coordinate system is the origin coordinate (0,0), the abscissa axis of the image coordinate system is horizontal, the maximum value is 1,400, the ordinate axis of the image coordinate system is vertical, and the maximum value is 1,040, i.e., the image width is 1400 × 1050.

It is assumed that the numerical matrix of the input image is *P*_0_ and the size is *P* × *Q*. In the scheme, 1400 × 1050 is used, and the numerical matrix *P*_0_ and the convolution kernel *M*_*k*1_ is convoluted:

(1) $$P_{1k}=P_{0}⊗M_{k1}$$

In the equation, ⊗ is the matrix convolution; *M*_*k*1_ is the *k*-th output in the first layer of the deep network, *k* = 1…256, i.e., the first layer consists of 256 feature convolution kernels, parameters of *M*_*k*1_ is obtained from VGG-16 model. *P*_1_*k*__ is the convolution output of the first layer in the deep network, with 256 outputs in total.

The convolution result of the first layer *P*_1_*k*__ is pooled. In the scheme, the maximum pooling method is adopted, i.e., every 2×2 local matrices in *P*_1_*k*__ are merged into one element, and the maximum value among the four elements is used as the result; the step size of pooling is 2. The pooling result *P*_1_*k*__ is *P*_1_*k*__ the size of *P*_1_*k*__ is 1/2 of the original size.

The convolution and pooling result of the first layer *P*_1*kc*_ is input to the second layer, and the result of the second layer is *P*_2*kc*_, the results of the l-1 layer *P*_(*l*−1)*kc*_ are sequentially obtained.

The operations of convolution and pooling of the first layer are obtained through recurrence:

(2) $$\begin{array}{r} P_{lk}=P_{\left(l-1\right)kc}⊗M_{kl} \end{array}$$

In the equation, *M*_*kl*_ is the convolution kernel matrix of the *k*-th feature of the first layer, the parameters *M*_*kl*_ are obtained. *P*_*lk*_ is the k-th output of the first layer in the deep network.

### Improvements in the Target Classification Algorithm

To accurately identify objects, the Hyper-Column scheme is used. The Hyper-Column scheme refers to a way to fuse the responses of neurons at various levels. It overcomes the information loss caused by multiple convolutions in the high-level convolution network, which makes the small target information in the high-level features be seriously missed and the detection effects on small targets are poor. Meanwhile, due to the addition of the underlying features, the bottom layer features have rich direction information, which can help the angle prediction (Capparelli et al., 2019; Toğaçar et al., 2020). Based on the original VGG-16 architecture, the 3rd, 4th, and 5th layers of the VGG-16 convolution layer are fused so that the model can have good detection robustness of smaller bottle objects on the conveyor belt; meanwhile, the information on the angle prediction of the object is increased by introducing a low-level neural response, thereby reducing the error of the angle prediction.

**Figure 3:**
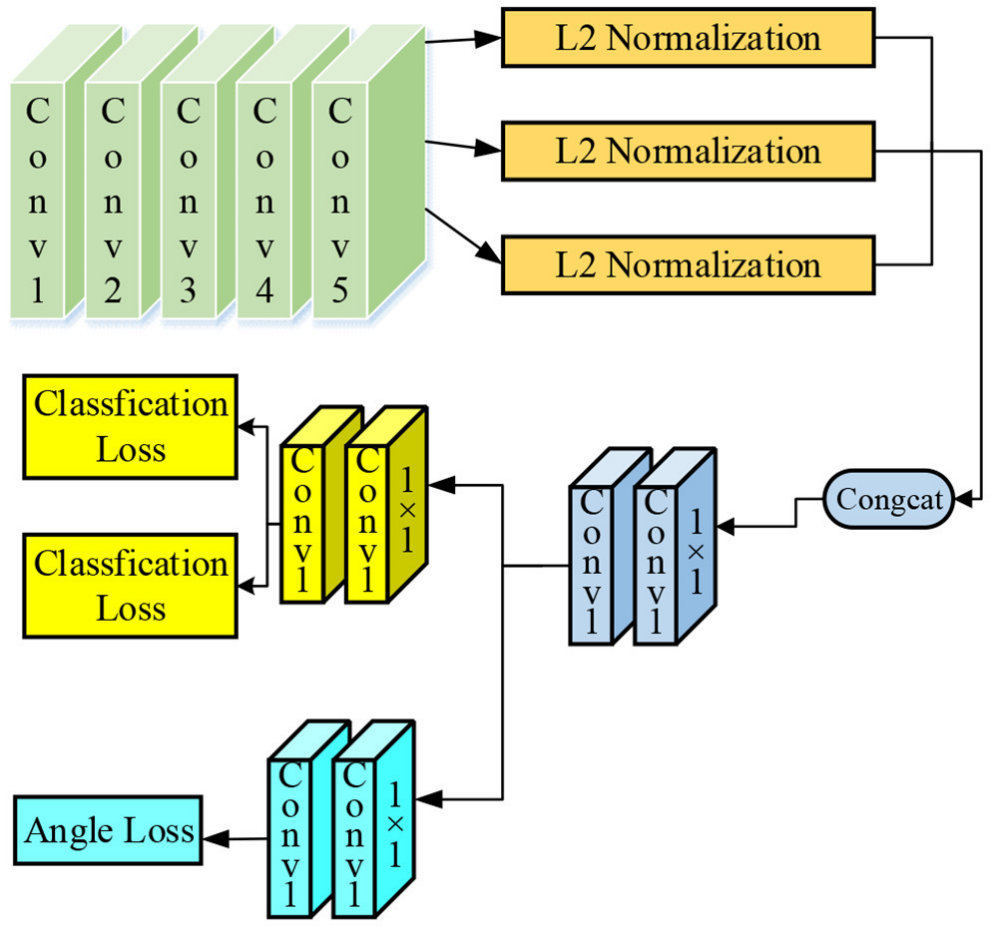
The improved VGG-16 classification model.

In summary, the following three improvements are made to the original VGG16 classification network: (1) the VGG-16 network structure with angle prediction is increased, and the response images of Conv3-4, Conv4-4, and Conv5-4 layers are fused to enhance the system performance through the feature fusion method; (2) the traditional classification regression loss is decoupled with angle prediction so that they are independently trained and predicted; and (3) the full connection layer is canceled, and the whole network is trained by using a full convolution network so that the network parameters are reduced to prevent over-fitting, and the training speed of the system is also improved. The improved VGG-16 classification model is shown in Figure 3.

## Experiment

To verify the correctness of the target recognition algorithm, in terms of the experimental method, the design adopts the method of pushing the input image, and the system performs the recognition and detection to verify whether the system can identify the target in the system. In the experiment, the bottle objects are used as the test objects.

### Verification of Fast R-CNN Algorithm

In the laboratory environment, 1,800 images are acquired and the collected data are grouped according to the ratio of the training set and test set, which is 9:1. The image resolution of images used in the test is 1400 × 1050. Hyperparameter settings are significant for CNN performance. Many optimization techniques can reduce the neural network model's hyperparameter setting difficulty and manually set hyperparameters. Table 1 shows the hyperparameter settings adopted in training to enable the CNN framework to obtain qualified prediction results.

**Table 1:** Parameter settings used in training in the laboratory environment.

**Item**	**Results**	**Item**	**Results**
Training set	1620 images (image resolution 1400 × 1050)	The test set	162 images (image resolution 1400 × 1050)
Learning rate	0.00114	Momentum	0.9
Number of anchor nodes	901	Weight-decay	0.0005
Dimension of anchor nodes	[600, 1000]	Iterations	Fine-tune: 40000 times
Number of images selected	VGG16 classification network: 2	NMS parameter	0.7
Number of Minni-batch in RPN	64	Detection threshold	0.99
The ratio of the prospect to the backdrop in RPN	[0, 0.3]	Flipped image	The first shift
Intersection-over-Union range of RPN prospect samples	No	Number of anchor nodes	901
Weight of criteria function	Position weight: 1; angle weight: 5	Training the updated layer	The 1st time: update the convolution layer and the FC layer; the 2nd time: update the FC layer. Shared convolution strategy applied

In the training process, the proportional selection of positive and negative samples will also have a great impact on training performance. In the task of detecting bottle-shaped objects, the identification of bottles is taken as the research object; thus, the samples are divided into two categories, i.e., bottles and non-bottles. Based on the above situation, the classified output layer of RPN and VGG-16 is set to 2 nodes, and the corresponding VGG-16 regression layer includes 4×2+1 = 9 nodes. The batch size of the RPN network is set to 64. Besides, the ratio of positive and negative samples is 1:1, which can ensure that the image has enough positive samples.

In the experiment, 1,800 images are used for the test, in which the total number of bottles is 260. During the measurement, whether the bottle is successfully detected is determined by the Intersection-over-Union (IoU) of the test result to the true position of the bottle being >0.5. The evaluation criteria used in the experiment are the false detection rate and the missed detection rate, which are defined as Equations (3) and (4), respectively:

(3) $$\begin{array}{r} \begin{array}{c} The\;false\;\mathrm{detection}\;rate=\\ \frac{number\;of\;false\;\mathrm{detection}\;cases}{number\;of\;t\arg ets\;\mathrm{detected}}\times 100\% \end{array} \end{array}$$

(4) $$\begin{array}{r} \begin{array}{c} The\;missed\;\mathrm{detection}\;rate=\\ \frac{number\;of\;missed\;\mathrm{detection}\;cases}{the\;total\;number\;of\;t\arg ets}\times 100\% \end{array} \end{array}$$

**Figure 4:**
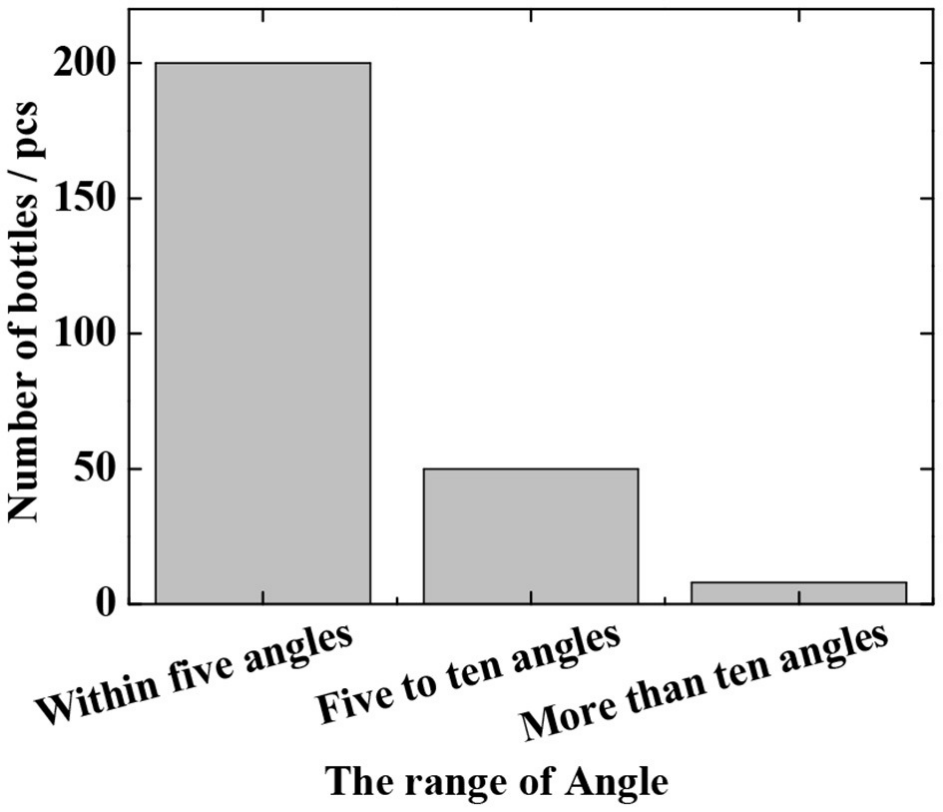
The angle detection result of the improved Fast R-CNN model in the laboratory environment.

The detection results of the Fast R-CNN algorithm are shown in Figure 4 and Table 2. As can be seen from Figure 4, 200 angles are detected within 5 degrees, 50 are detected from 5 degrees to 10 degrees, and 8 are detected above 10 degrees. It can be seen from the results that in the laboratory environment, the predicted angle of the bottle-shaped article is predicted accurately, and the data of Table 2 shows that the false detection rate and the missed detection rate are 3.2 and 8.7%, respectively. In terms of the detection time, since the CNN network itself has a long forward propagation time, the time is 220 ms.

**Table 2:** The detection result of the improved Fast R-CNN model in the laboratory environment.

**Average error**	**Standard deviation**	**Threshold**	**False detection rate/%**	**Missed detection rate/%**	**Time consumption**
7.70	13.56	250	3.3	8.9	210 ms

**Figure 5:**
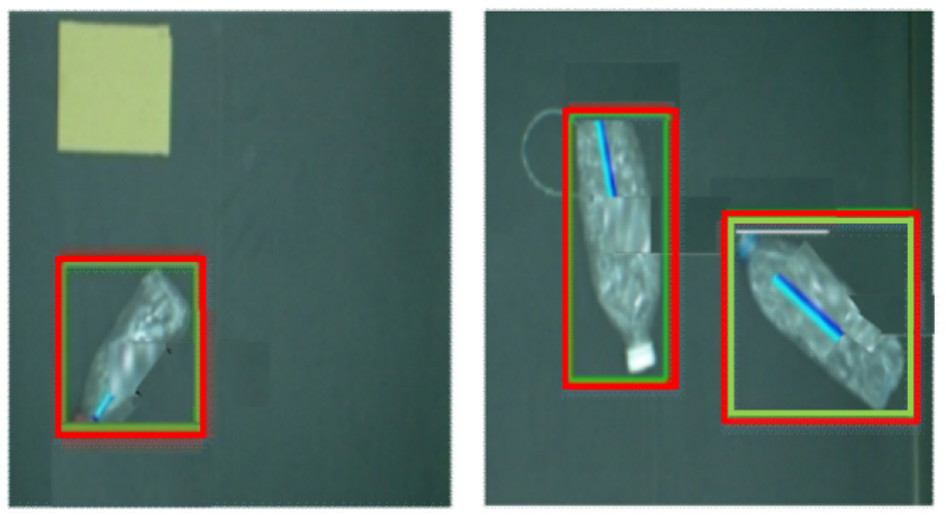
Physical graph of the test in the laboratory environment.

Figure 5 shows the detected images of objects in the laboratory environment. As can be seen from Figure 5, the Fast R-CNN algorithm can accurately detect the position and angle of the bottle.

The results in the laboratory environment show that the improved algorithm model of deep learning can accurately predict the position and angle of the bottle, which meets the needs of industrial robot target recognition.

### Verification of VGG-16 Classification Network Model Algorithm Based on Hyper-Column Scheme Improvement

The above simulation verification is carried out in the experimental environment. To verify the target recognition effect of the improved VGG-16 classification network based on the Hyper-Column scheme, field verification is carried out through collecting 1,600 bottles in the real environment from a waste treatment plant in Xi'an City, Shaanxi Province. Similar to the above method, a 9:1 training test ratio is used, in which the total target number of the bottle is 160. The parameter settings for the training phase are shown in Table 3.

**Table 3:** Parameter settings used in training in the real environment.

**Item**	**Results**	**Item**	**Results**
Training set	1440 images (image resolution 1600 × 1200)	The test set	160 images (image resolution 1600 × 1200)
Learning rate	0.00114	Momentum	0.9
Number of anchor nodes	901	Weight-decay	0.0005
Dimension of anchor nodes	[600, 1000]	Iterations	Fine-tune: 40,000 times
Number of images selected	VGG16 classification network: 2	NMS parameter	0.7
Number of Minni-batch in RPN	64	Detection threshold	0.99
The ratio of the prospect to the backdrop in RPN	[0,0.3]	Flipped image	No
Intersection-over-Union range of RPN prospect samples	No	Number of anchor nodes	901
Weight of criteria function	Position weight: 1; angle weight: 5	Training the updated layer	The 1st time: update the convolution layer and the FC layer; the 2nd time: update the FC layer. Shared convolution strategy applied

**Figure 6:**
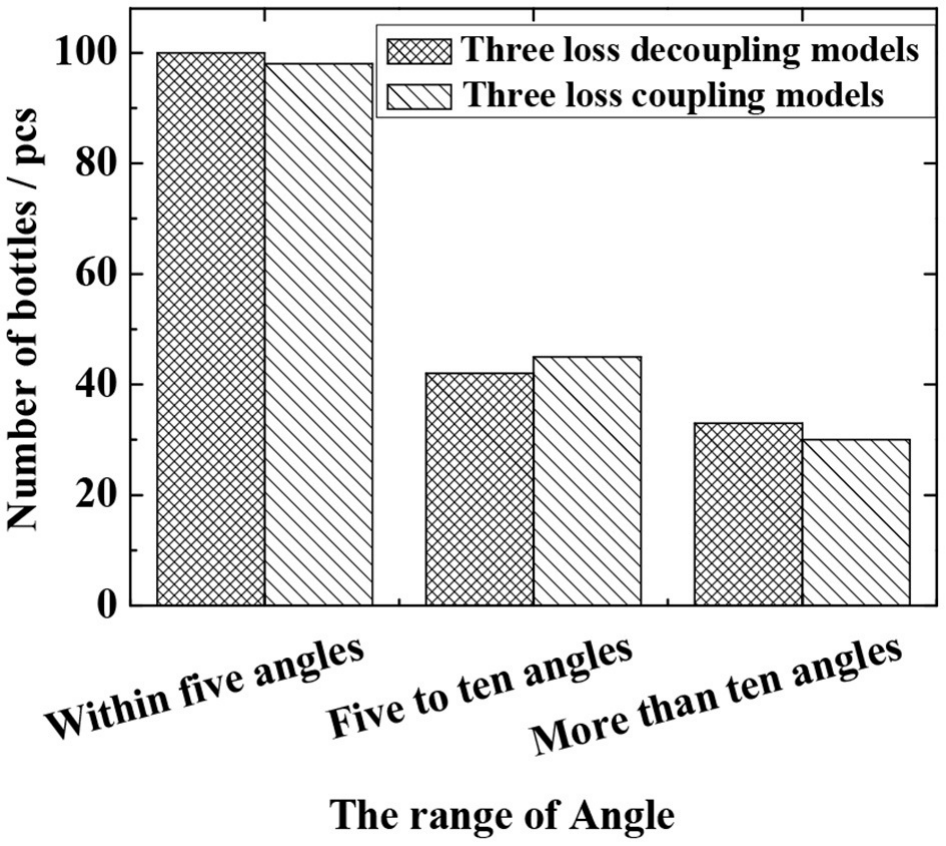
The angle detection result of the improved Fast R-CNN model in the real environment.

In the experiment, a total of 1,600 images are tested, in which the total number of bottles is 204 (some images have no bottles, and the crossover threshold is 0.5). The evaluation criteria are the false detection rate and the missed detection rate (as defined in section Verification of Fast R-CNN Algorithm), and the test results are shown in Figure 6 and Table 4. It can be seen that the actual missed detection rate and false detection rate are high due to the poor image pixels captured in the real environment. As shown in Figure 6, in the decoupling model, 100 are detected for angles within 5 degrees, 42 for angles between 5 and 10 degrees, and 33 for angles >10 degrees. In the coupled model, 98 angles are detected to be within 5 degrees, 45 are detected between 5 and 10 degrees, and 30 are detected above 10 degrees. In the meantime, the results of coupling and decoupling models are compared, and the results are summarized in Table 4. The three loss decoupling models can provide lower false detection rate and missed detection rate, thereby obtaining more accurate detection results.

**Table 4:** The detection result of the improved Fast R-CNN model in the real environment.

**Model**	**Average error/degree**	**Standard deviation/degree**	**Numbers within the threshold**	**False detection rate/%**	**Missed detection rate/%**	**Time consumption**
3 loss coupling model	7.71	13.53	176	7.4	21.3	230
3 loss decoupling model	6.95	11.41	174	5.8	19.4	260

**Figure 7:**
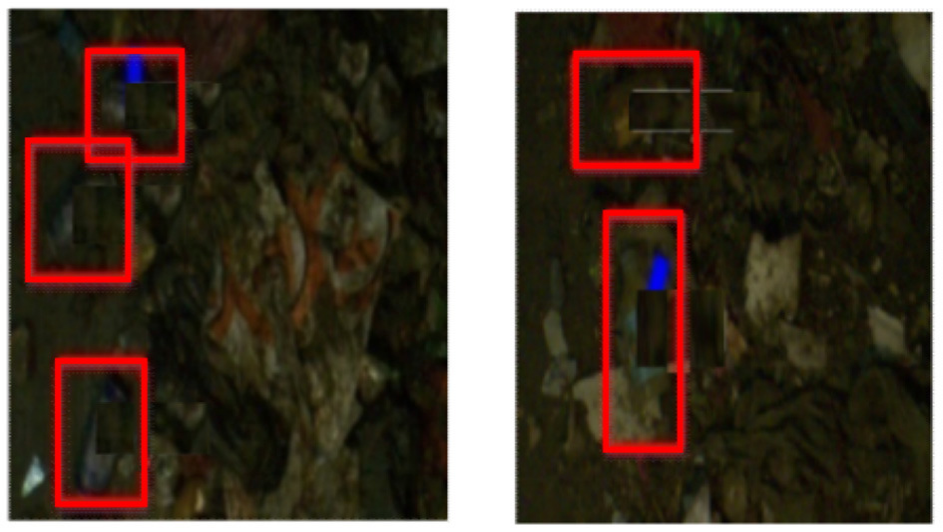
Physical graph of the test in the real environment.

Figure 7 shows the test results in the real environment. It can be seen that even in the case of poor image quality (even difficult to distinguish by the naked eye), the algorithm can still obtain better detection results.

The test results of the real waste treatment plant images show that the improved Hyper-Column-based VGG-16 model can better detect the position and angle of the bottle, which meets the task requirements.

### Comparison of the Improved VGG-16 Classification Network Model Based on the Hyper-Column Scheme and Other Advanced CNNs

The VGG-16 classification network model improved based on the Hyper-Column scheme is verified on the Matlab network simulation platform. The NYU Depth V2 database is introduced (Chen et al., 2019) with the leave-one-out evaluation method adopted; that is, the dataset with a sample space of 1100 is divided into subsets of 1000 and 100, with the subset of 1000 used as the training set and subset of 100 as the test set. The constructed system model is compared with advanced CNNs (AlexNet, GoogleNet, LeNet, ZF-Net, and ResNet) (Wang et al., 2017; Fadlullah et al., 2018; Luo et al., 2019; Hosny et al., 2020; Wang and Jia, 2020). The following equation shows the accuracy.

(5) $$\begin{array}{r} ACC=\frac{TP+TN}{TP+TN+FP+FN} \end{array}$$

In Equation (5), *TP* represents a positive sample with positive prediction, *FP* represents a negative sample with positive prediction, *FN* represents a positive sample with negative prediction, and *TN* represents a negative sample with negative prediction.

**Figure 8:**
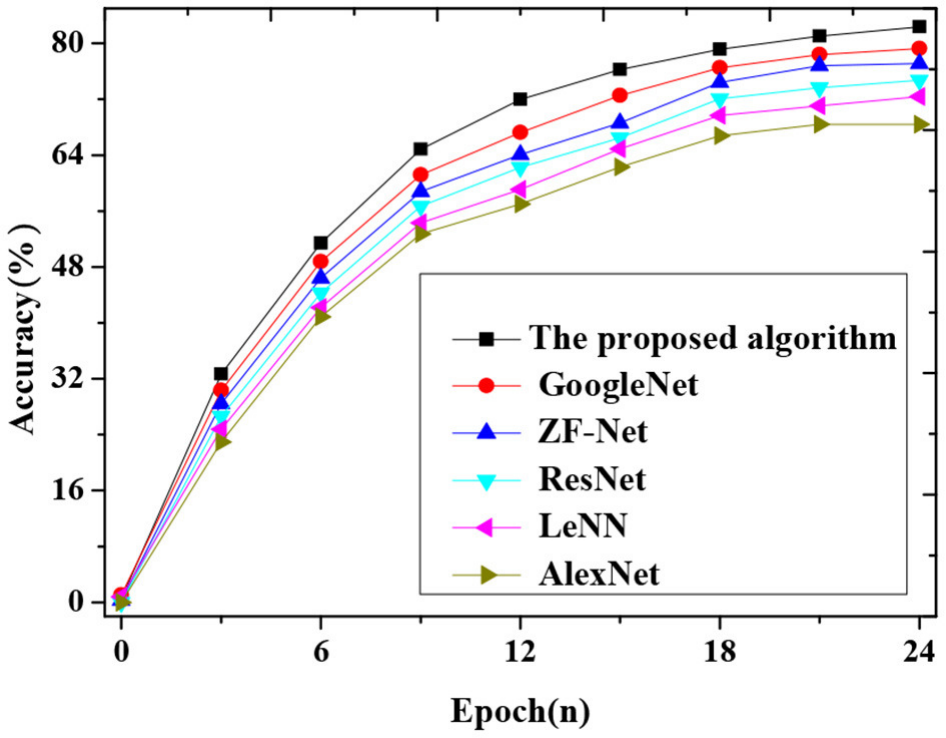
Accuracy curves compared with advanced CNNs.

Figure 8 shows the accuracy comparison between the improved algorithm and other advanced neural networks.

Figure 8 shows the accuracy comparison of the improved algorithm with other advanced neural networks. The result shows that the improved algorithm's accuracy reaches 82.34%, at least 3% higher than other advanced CNN algorithms such as AlexNet, GoogleNet, LeNet, ZF-Net, and ResNet.

## Discussion

### Comparison of the Results of Fast R-CNN Algorithm and Improved Network Model Based on Hyper-Column Scheme

The detection effects of the Fast R-CNN algorithm on the detection of bottle-like objects in the laboratory environment and the detection effects of the VGG-16 algorithm based on the Hyper-Column scheme on detecting complex bottled objects in a realistic and complex environment are verified. The experimental results show that the Fast R-CNN algorithm can accurately detect bottled objects under the experimental environment, and the distribution of detection rate and the error rate is <10%. The improved VGG-16 algorithm based on the Hyper-Column scheme can accurately detect bottled objects in a complex real-world environment with a missed detection rate of <10% and an error rate of <30%. Both algorithms have better positioned and identified the target objects. In comparison, the Fast R-CNN algorithm in the experimental environment can complete the detection of the target item in 0.2 s, while the VGG-16 algorithm based on the Hyper-Column scheme can complete the detection in 0.24 s in the 3 loss coupling models, and the 3 decoupling models are completed in 0.28 s. Although the false detection rate and the missed detection rate of the 3 loss decoupling models are small, they are slightly inferior to the 3 loss coupling models in terms of detection consumption time. The constructed algorithm's accuracy is compared with that of other advanced neural networks. The analysis shows that the constructed algorithm's accuracy reaches 82.34%, at least 3% higher than other advanced CNN algorithms such as AlexNet, GoogleNet, LeNet, ZF-Net, and ResNet. The reason may be that the original DL algorithm's feature fusion and the training speed increase improve industrial robot target recognition accuracy.

### Results Discussions

The applications of the Fast R-CNN algorithm and the improved Hyper-Column-based VGG-16 classification network in industrial robot target recognition and classification are explored. The experimental results show that deep learning plays an important role in the visual recognition of industrial robots. Compared with the previous research, the improvement of the algorithm makes the industrial robots convolve and pool in the visual recognition, leading to the results that the average false detection rate is <5.5% and the average missed detection rate is 17%. The test results have shown the high precision and high accuracy of the algorithm. The deep learning algorithm is suitable for industrial applications. Meanwhile, it also shows that artificial regularization can improve the learning and recognition of the algorithm. It can be seen from the experimental results that the algorithm model proposed in the experiment can meet the requirements of modern industry for industrial robot vision works.

## Discussion

Artificial intelligence has superior feature extraction performance and great development potential in the industry 4.0 era. Therefore, to solve these problems, the image layers are convolved and pooled through the deep learning model of artificial intelligence, and the visual recognition system of industrial robots is optimized through the advanced methods of the target classification algorithm. In the aspect of visual recognition, the recognition algorithm of target objects in a complex background environment is studied. The results have shown that both the Fast-RCN algorithm and the improved VGG-16 classification network based on the Hyper-Column scheme can complete the localization and recognition of the target objects, indicating that the Fast-RCN algorithm and the Hyper-Column-based scheme have improved the accuracy and effectiveness of target recognition and positioning of VGG-16 classification network.

At present, the research on industrial robots in artificial intelligence has yet to be improved in China. The application of deep learning in artificial intelligence to the visual system of industrial robots has achieved preliminary results through the experiment. If the relevant algorithms of artificial intelligence can be applied in the research, development, and production process of industrial robots in future research, it will certainly promote the industrial robot technology to develop more rapidly. There are many algorithms in the deep learning theory of artificial intelligence, which can be used to solve a series of problems that occurred in the development of industrial robots. With the rapid development of communication technology, the 5G era has arrived. The new generation of wireless communication technology not only means faster transmission speed but also better security performance for industrial robots. The application of these intelligent algorithms to the development of industrial robots with higher service levels will bring new opportunities for the development of industrial robots.

## Data Availability Statement

The raw data supporting the conclusions of this article will be made available by the authors, without undue reservation.

## Author Contributions

All authors listed have made a substantial, direct and intellectual contribution to the work, and approved it for publication.

## Conflict of Interest

LLi was employed by Huawei Technologies Co. Ltd. The remaining authors declare that the research was conducted in the absence of any commercial or financial relationships that could be construed as a potential conflict of interest.
